# Association between Smoking and Noise-Induced Hearing Loss: A Meta-Analysis of Observational Studies

**DOI:** 10.3390/ijerph17041201

**Published:** 2020-02-13

**Authors:** Xiaowen Li, Xing Rong, Zhi Wang, Aihua Lin

**Affiliations:** 1 Department of Medical Statistics, School of Public Health, Sun Yat-sen University, Guangzhou 510080, China; 2 The Institute of Occupational and Environmental Health, Guangzhou Medical University, 1 Tianqiang St., Huangpu West Ave., Guangzhou 510620, China; 3 Department of Health Service and Management, Xinhua College of Sun Yat-sen University, Guangzhou 510520, China

**Keywords:** noise-induced hearing loss, smoking, meta-analysis, dose-response relationship

## Abstract

The purpose of this study was to synthesize the results of previously published observational studies through meta-analysis to clarify the association between smoking and noise-induced hearing loss (NIHL). We searched several databases as of October 2019. Based on the results of heterogeneity analysis (*Q* statistic and *I*^2^ statistic), a fixed effect model (for no heterogeneity; *Q* test *P* > 0.1 and *I*^2^ ≤ 50%) or a random effects model (for heterogeneity) was used to calculate the pooled odds ratios (ORs). We explored the potential dose-response relationship between smoking and NIHL as well. In total, 27 studies involving 30,465 participants were included. Compared with non-smokers, the pooled OR of current smokers was 2.05 (95% Confidence interval (CI): 1.71–2.46), and of former smokers was 1.11 (95% CI: 1.05–1.18). We found a curve linear association between an increasing number of pack-years (packages/day × smoking years) and risk of NIHL. The dose-response meta-analysis suggested that when the number of pack-years was less than fifteen, the risk of NIHL was increasing, and the highest combined OR was 5.25 (95% CI: 2.30–11.96) for pack-years of fifteen. After fifteen pack-years, the pooled OR had a slow decline. Our study indicated that smoking is a risk factor for NIHL. Current smokers have a higher risk than former smokers, and there is a positive dose-response relationship between smoking and NIHL.

## 1. Introduction

Noise-induced hearing loss (NIHL) is chronic and irreversible sensorineural hearing loss resulting from long-term exposure to noise. It affects the daily social life of patients and brings a huge burden on society and economy. According to the WHO, around 466 million people worldwide suffer from disabling hearing loss, and it is estimated that unaddressed hearing loss poses an annual global cost of US$750 billion [1]. About 16% of the world’s disabling hearing loss is caused by noise exposure in the workplace [2], and NIHL has become one of the most important work-related diseases around the world. In addition, it is estimated that nearly 600 million workers worldwide have a history of occupational noise exposure [3]. 

NIHL is related to multiple factors. In addition to occupational noise, other factors (such as organic solvent [4], high-temperature [5], no use of hearing protection device [6], alcohol [7], genes [8], comorbidity [9], etc.) may be independent factors or have a synergistic effect with noise to increase the risk of NIHL. Smoking is a risk factor for many illnesses, and many published studies [10,11,12,13] have suggested that it may also be associated with NIHL. Some toxic and harmful substances like nicotine from tobacco burning may affect hearing. However, smokers are widely distributed all over the world, especially in China, with an estimated more than 300 million people (one third of the total number of smokers worldwide) [14]. As a lifestyle that is one of the leading preventable causes of death but difficult to quit, the association between smoking and NIHL has drawn increasing attention. Although a meta-analysis [15] has concluded that smoking is associated with hearing loss, prior analyses focused on people without occupational noise exposure, and the pathogenesis of NIHL is different from other hearing loss. Therefore, we conducted a meta-analysis of observational studies to assess the relationship between smoking and NIHL in noise exposed workers.

## 2. Materials and Methods

### 2.1. Literature Search Strategy

The meta-analysis was performed in accordance with the PRISMA guidelines [16]. We conducted a literature search in Pubmed, Embase, Web of Science, Scopus, Wanfang, and CNKI databases for studies published in English and Chinese up to October 2019. The search terms were NIHL and smoking with their synonyms (noise induced hearing loss or noise induced deafness or noise deafness) AND (smoke or smoker or smoking or cigarette or tobacco or cigar). We also reviewed the reference lists of retrieved articles for other pertinent papers.

### 2.2. Inclusion and Exclusion Criteria

The inclusion criteria were as follows: (1) the study design was a cohort, case-control or cross-sectional; (2) study population had a history of occupational noise exposure; (3) NIHL was clearly defined as the outcome; (4) study provided odds ratio (OR) or relative risk (RR) with the corresponding 95% confidence interval (CIs). If a study was published in multiple papers, we included only one with sufficient information. Review, conference, or experimental articles were excluded. 

### 2.3. Data Extraction and Quality Assessment

Two investigators (X.L. and X.R.) independently extracted the following information from eligible studies: first author, year of publication, country, source of participants, study design, sample size, age, gender, diagnostic of NIHL, smoking information, adjusted OR/RR with 95% CIs, and adjusted or matched variables. Disagreements were solved through discussion.

We used the Newcastle-Ottawa Scale [17] to assess the quality of cohort or case-control studies. The judgement was based on three areas: selection of participants, comparability of groups, and exposure/outcome ascertainment. Scores ranging from 0 to 9 reflect an improvement quality of studies. For cross-sectional studies, an 11 items checklist recommended by Agency for Healthcare Research and Quality (AHRQ) [18] was applied. Articles scoring 0–3 points, 4–7 points, and 8–11 points were classified as low, moderate, and high-quality studies.

### 2.4. Statistical Analysis

OR was used as a measure of the association between smoking and NIHL. Due to the low incidence of NIHL, the reported RR was approximately considered as OR. When smoking status was just divided into smokers and non-smokers, smokers were defined as current smokers. In addition, when OR was reported separately at different smoking levels, we extracted the highest level of results. Two articles [19,20] separately estimated OR and 95% CI in two levels of noise exposure, and they were treated as different studies in the analysis.

Before calculating the overall pooled OR, we used *Q* test and *I*^2^ statistic to quantify the heterogeneity of studies. If *P* value for *Q* test was more than 0.10 and *I*^2^ value was less than 50%, we used a fixed effect model. Otherwise, a random effects model was applied [21]. And we did subgroup analyses according to study design (cohort vs. case-control vs. cross-sectional), gender (both vs. male vs. female), mean age (<40 vs. ≥40), race (Mongoloid vs. Caucasian vs. others), quality of studies (high quality vs. moderate quality), number of adjusting variables (0 vs. ≥1) and publication year (<2010 vs. ≥2010). The race was roughly classified on the basis of the country reported in the study. People living in China, Japan, Malaysia, Indonesia, Brunei, and Nepal were seen as Mongoloid; Caucasians were from the United States (USA), Britain, Italy, Iran, Denmark, Switzerland, and Germany; Brazilian was classified separately. 

In addition, a dose-response analysis of pack-years and NIHL was estimated. Pack-years is a measure of the amount of cigarettes a person has smoked over an extended period, which is equal to the number of packages (/20 cigarettes) per day multiplied by the smoking time (/years). For example, smoking one package every day for two years equals to two pack-years. Articles that provided at least three quantitative categories were included in this calculation. Since all studies reported dose in groups, we assigned the midpoint of the group range as the dose value, and for the highest open-ended group, multiplied the lower limit by 1.5 times. We evaluated the potential curve liner relationship between the number of pack-years and NIHL by using restricted cubic splines with three knots (10%, 50%, and 90%) of the distribution [22,23]. We tested whether the coefficient of the second spline is equal to zero to determine whether the relationship is linear or non-linear. A coefficient not equal to zero for the second spline indicates a non-linear relationship [24].

A sensitivity analysis was also conducted to see the influence on the overall result by omitting each study. In addition, we recalculated the pooled OR after omitting those articles with extremely high ORs (>10). We used Begg’s rank correlation test and Egger’s linear regression test to assess potential publication bias [25,26]. If publication bias was indicated, further trim-and-fill method [27] was used to recalculate the pooled OR. All statistical analyses were performed using R version 3.6.0 (R Foundation for Statistical Computing, Vienna, Austria). For all statistical tests other than Q tests, which have different statistical significance criteria as described above, a two-sided *P*-value < 0.05 was considered statistically significant. 

## 3. Results

### 3.1. Literature Search

**Figure 1:**
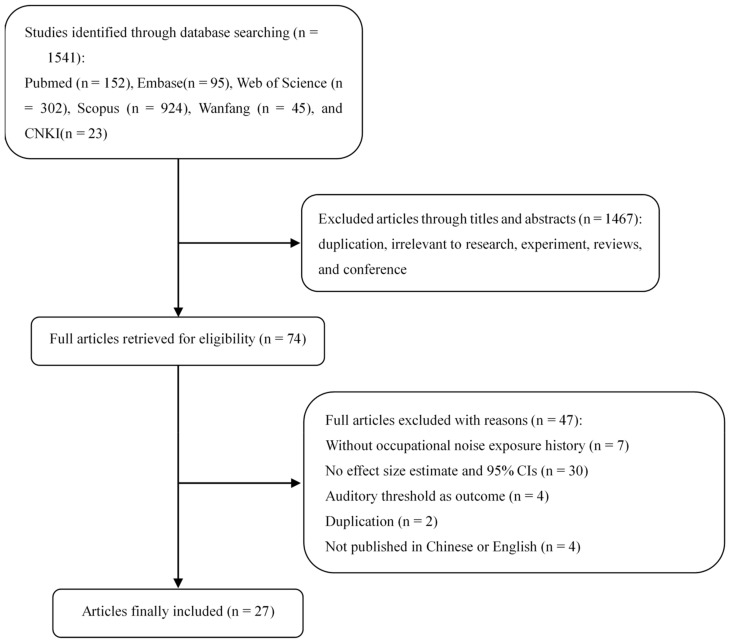
Literature search and selection flowchart.

Based on the database and search terms, we obtained 1541 articles to screen. Through browsing titles and abstracts of papers, we excluded 1467 records. After retrieving and reviewing 74 full articles, we excluded 47 records. Seven studies were excluded because participants in the research didn’t have a history of occupational noise exposure. A further 30 studies were excluded because they didn’t provide effect size estimate and 95% CIs to calculate the pooled OR. 4 studies were excluded because they used auditory threshold as outcome. Two studies were excluded because results were repeatedly reported in other articles. Four studies not published in Chinese or English were also excluded. Our meta-analysis includes 27 studies [7,10,11,12,13,19,20,28,29,30,31,32,33,34,35,36,37,38,39,40,41,42,43,44,45,46,47] in this meta-analysis. Selection details are shown in Figure 1. There were four cohort studies, two case-control studies, and 21 cross-sectional studies.

### 3.2. Characteristics of the Included Studies

Table 1 shows the main characteristics of 27 studies included in our meta-analysis. Studies were published between 1987 and 2018. And a total of 30,465 workers were included in our review. Among all included studies, 18 studies were from Asia; 5 studies were from Europe; 3 studies were from North America, and 1 study was from South America. The diagnostic criteria showed differences among these studies; 16 studies were based on speech frequency, and 11 studies were based on high frequency. In addition, the quality assessment scores for the 4 cohort studies were in the range of 6–8, and the average score was 7.25 points. For two case-control studies, the scores were 7 and 8 points. There were 21 cross-sectional studies with the scores ranging from 6 to 10, and the average score was 8.14 points.

### 3.3. Association between Current Smokers and Risk of NIHL

**Figure 2:**
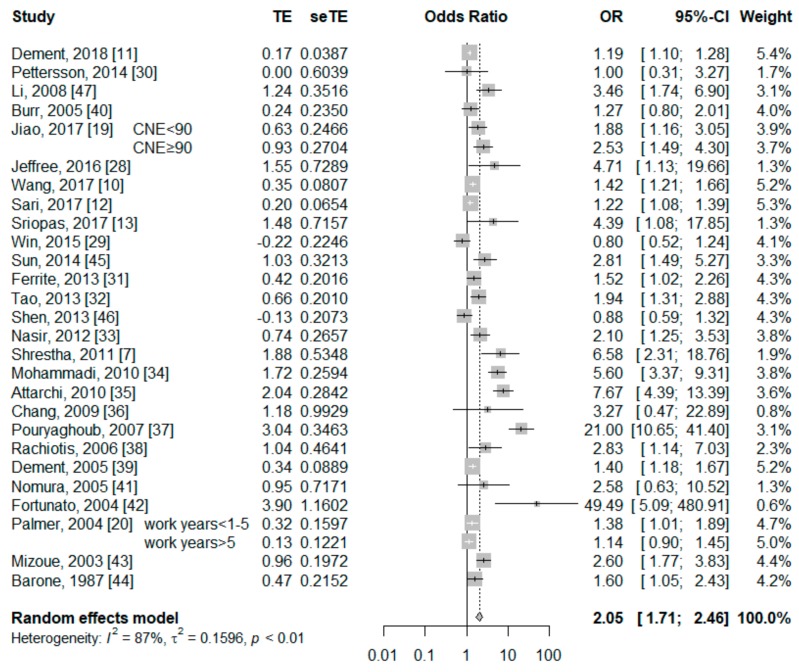
Forest plot for the association between current smokers and NIHL risk.

Of the 27 included articles, 29 studies (2 articles reported OR separately for different noise exposure history) assessed the association between current smokers and risk of NIHL. Among the 29 studies, 20 reported a positive relationship between current and risk of NIHL, while nine found no association. Figure 2 shows the results of pooled OR by a random effects model. The pooled OR of NIHL for current smokers was 2.05 (95% CI: 1.71–2.46) with a significant heterogeneity across studies (*Q* test *P* < 0.001, *I*^2^ = 87%).

### 3.4. Association between Former Smokers and Risk of NIHL

Six studies (1 articles reported OR separately for different noise exposure history) provided information on the association between former smokers and risk of NIHL. Supplementary Figure S1 shows the results of pooled OR from a fixed effect model. Of the six included studies, only one showed a positive relationship, while others suggested no statistical significance. The pooled OR of NIHL for former smokers was 1.11 (95% CI: 1.05–1.18). No heterogeneity was detected (*Q* test *P* = 0.394, *I*^2^ = 4%).

### 3.5. Subgroup Analyses

Table 2 shows the results of subgroup analysis for current smokers and NIHL risk. Study design, gender, mean age, race, quality of studies, number of adjusting variables and publication year were conducted in the subgroup analysis. Overall, the results for most subgroups indicate a positive relationship between current smokers and risk of NIHL. According to the study design, the main heterogeneity came from cross-sectional studies (*Q* test *P* < 0.001, *I*^2^ = 89%), and for cohort studies and case-control studies, there was no evidence of heterogeneity (*Q* test *P* = 0.504, *I*^2^ = 0%; *Q* test *P* = 0.418, *I*^2^ = 0%). According to gender, the pooled OR was 3.05 (95% CI: 1.90–4.89) for male, 1.50 (95% CI: 1.28–1.76) for both, and 1.52 (95% CI: 1.03–2.27) for female. According to race, the pooled OR was 1.88 (95% CI: 1.50–2.36) for Mongoloid, 2.41 (95% CI: 1.70–3.42) for Caucasian, and 1.52 (95% CI: 1.03–2.27) for others. According to the number of adjusting variables, the pooled OR was 1.58 (95% CI: 0.86–2.90) for 0, and 2.18 (95% CI: 1.77–2.69) for ≥1.

### 3.6. Dose-Response Analysis

**Figure 3:**
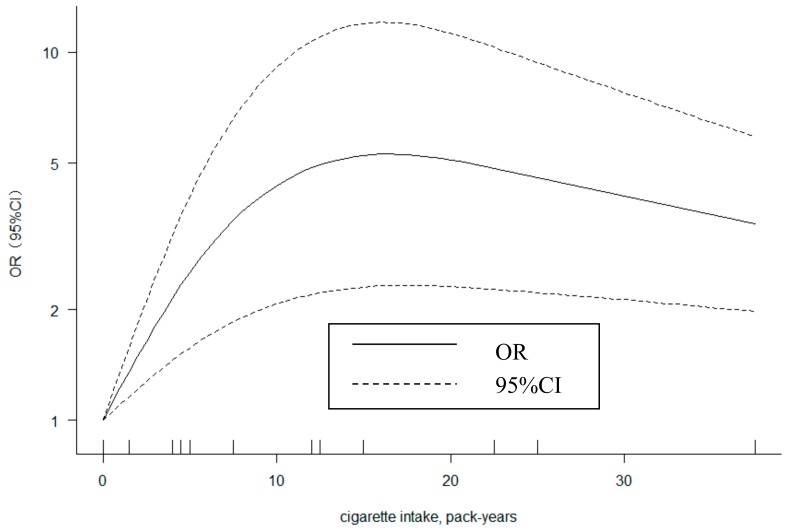
Dose-response relationships for cigarette intake pack-years and NIHL.

For the dose-response analysis, a total of 8 studies were included based on our inclusion criteria. The 8 included studies reported data for pack-years of cigarette intake and risk of NIHL. As Figure 3 shows, we observed evidence of non-linear association based on the restricted cubic splines with the random effects model (*P* < 0.001). There was a non-linear increase in risk of NIHL with increasing of pack-years (about or less than 15). Compared with non-smokers, the estimated ORs of NIHL were 2.53 (95% CI: 1.58–4.05) for five pack-years of cigarette intake, 4.34 (95% CI: 2.07–9.11) for 10 pack-years, 5.25 (95% CI: 2.30–11.96) for 15 pack-years, 5.10 (95% CI: 2.31–11.27) for 20 pack-years, 4.56 (95%CI: 2.22–9.38) for 25 pack-years, 4.06 (95% CI: 2.13–7.77) for 30 pack-years, and 3.62 (95% CI: 2.03–6.46) for 35 pack-years. We found a substantial heterogeneity across studies (*Q* test *P* < 0.001, *I*^2^ = 92.8%).

### 3.7. Sensitivity Analysis and Publication Bias

The results were not significantly different after omitting two studies [37,42] with extremely high ORs. The sensitivity analysis for current smokers and NIHL risk showed that the result was not significantly affected by removal of any one study. Nevertheless, among studies for former smokers, sensitivity analysis hinted that, omitting the study by Dement [11], the pooled OR would change to 1.18 (95% CI: 1.00–1.39) (Supplementary Figure S2). 

Supplementary Figure S3 shows an asymmetric funnel plot of studies researching the relationship between current smokers and NIHL. It indicated a potential publication bias. In addition, the Begg’s rank correlation test and the Egger’s linear regression test both confirmed potential publication bias (*P* = 0.007; *P* < 0.001). In view of this, we used trim-and-fill method to recalculate the pooled OR. The result was 1.34 (95% CI: 1.10–1.64), which still indicated the same positive association. Publication bias about former smokers and NIHL was not found by either the Begg’s test or the Egger’s test (*P* = 0.091; *P* = 0.173).

## 4. Discussion

In this meta-analysis, we confirmed the hypothesis that smoking is associated with increased risk of NIHL. Both current smokers and former smokers had a higher risk of NIHL than non-smokers. And we found a dose-response relationship between smoking and NIHL.

However, the specific mechanism of smoking and NIHL is unclear. Nicotine and other substances in tobacco may have ototoxicity, damaging cochlear hair cells by increasing carbon monoxide hemoglobin or reducing the volume of cochlear blood flow [48,49]. In addition, experimental studies have found nicotine-like receptors in hair cell, suggesting that nicotine has a direct ototoxic effect on hair cell function [50]. Smoking may be an independent risk factor for NIHL. Some studies [37] found that the combined effect of smoking and occupational noise was comparable to the sum of the independent effects of each factor. However, other studies [47] indicated that smoking and noise might have a synergistic effect on NIHL. 

Our study showed that the pooled OR for current smokers was higher than OR for former smokers, indicating that quitting smoking could reduce the risk of NIHL. It may be associated with the dose-response relationship between smoking and the risk of NIHL risk; former smokers have lower exposure to smoke. We performed a dose-response analysis based on eight eligible studies. It showed a non-linear relationship between cigarette intake pack-years and NIHL. The OR and its 95%CI of each dose are always greater than one. The general trend also showed that the risk of NIHL would increase with the smoking dose increasing. The slow decline in the fitting model may be due to the fact that the dose concentration of the included studies was mainly between 0 and 15 pack-years. In the total of 19 dose points, only three points were larger than 15, which made the latter trend less accurate. In addition, the studies included in the dose-response analysis were mainly cross-sectional studies. There might be a healthy worker effect in the population. Therefore, a smoking cessation program is important for workers in a noise exposure environment.

In the subgroup analysis, we found that heterogeneity mainly came from cross-sectional studies. It is known that the results of cohort and case-control studies are more reliable. But there were no differences in results between the three types of studies. The subgroup analysis by gender showed that the positive association between smoking and NIHL was stronger in male than female. Because the smoking group of women was small, we only included one study focusing on female participants. And in general, men smoke more than women. Further, in the working environment, men generally have a higher noise exposure dose and a longer duration. Some studies suggested that men were more likely to suffer from NIHL as well [51,52,53]. According to subgroup analysis of race, the OR of current smoking was the highest in the Caucasian population. It may be attributed to genetics, and the previous study indicated that white people were more susceptible to NIHL [54]. Since some cited countries are multi-ethnic, and there are some country-level factors (such as country-specific industry standards for allowable noise levels), the method of simply classifying races based on the country is rough, so the results have some limitations. With regard to number of adjusting variables, group 0 didn’t show a positive association. It might attribute to those unadjusted variables (such as age) overwhelming the certain effect of smoking. 

Egger’s linear regression test and Begg’s rank correlation test for the study of current smokers and NIHL were both suggestive of publication bias, which may be related to the inclusion of articles included published in Chinese and English only. In the process of paper screening, four studies published in other languages were eliminated. There was no search for unpublished grey documents. To address the potential of publication bias, we conducted the trim-and-fill method to adjust the influence of publication bias. There was no substantial change, suggesting that the result was not affected by this bias.

Despite previous meta-analysis [15] suggesting that smoking may increase the risk of hearing loss, this study was the first meta-analysis study to explore the relationship between smoking and NIHL. In our study, we focused exclusively on workers with a noise exposure history. Although there was high heterogeneity, subgroup analysis and sensitivity analysis suggested that our results were robust. And a dose-response analysis of pack-years and NIHL was also carried out to assess the dose-response relationship between them.

There are still some limitations in the present study. First, due to the lack of cohort and case-control studies, we added cross-sectional studies. However, cross-sectional studies have a selection bias due to the defects of its design, so that the results are not as reliable as the other two study designs. Secondly, the diagnostic criteria of NIHL were entirely different in each study. Some chose high-frequency (3 kHz) hearing loss as the standard, and some were based on speech frequency (−2 kHz) hearing loss. Moreover, the frequency selections of hearing thresholds were varied as well, which might lead to the heterogeneity. Third, there are also significant differences in the correction of factors that may affect the relationship between smoking and NIHL. The previous studies may not have adjusted all of the confounding factors similarly, such as heredity, with a great effect on NIHL. Fourth, in the dose-response analysis, few of included studies had high dose results, which affected the stability of the rear part of the curve.

According to the shortcomings of our research, we hope to have more long-term follow-up prospective cohort studies to explore the relationship between smoking and NIHL to further confirm our conclusions. They also need uniform diagnostic criteria. In addition, the synergistic effect between smoking and noise interests us. We look forward to seeing a subgroup analysis of diverse occupational noise exposure history. Based on the available information, we have reason to believe that smoking is a risk factor for many diseases, and it can affect workers’ hearing health. Relevant departments can provide some smoking cessation programs in the occupational noise environment, especially for men. The Government can also reduce tobacco consumption by increasing tobacco taxes.

## 5. Conclusions

In summary, our study indicated that smoking is a risk factor for NIHL. Quitting smoking can reduce the risk of NIHL. There is a non-linear dose-response relationship between the number of smoking pack-years and NIHL. When the dose is less than 15, the risk will add over the increase of pack-years.

## Figures and Tables

**Table 1:** Main characteristics of 28 included studies on smoking and NIHL risk.

First Author and Year	Country	*n*	Mean Age/Age Range	Gender	Smoking Information	Diagnostic Criteria of NIHL	Adjusting or Matching Variables	Quality Assessment
Cohort study								
Dement, 2018 [11]	USA	4275	59.2	Both	Never smoked, Past smoker, Current smoker, Smoking	Index weighted average threshold >25 dB at 1, 2, 3, and 4 kHz	Age, race, sex, organic solvent exposure, duration of trade work, loud or very loud noise exposure, hypertension	8
Pettersson, 2014 [30]	Sweden	184	NA	Male	Smoker, Non-smoker	>30 dB at 0.5 kHz or >25 dB at 1–2 kHz and >25 dB for at least one of the frequencies of 3, 4, 6 kHz	VWF in the right hand, age, exposure	8
Li, 2008 [47]	China	408	26.5	Both	Smoking number per day: 0, −10, 10–20, >20	Hearing any tone >25 dB	Cumulative noise exposure (CNE)	6
Burr, 2005 [40]	Denmark	1237	18–59	Both	Never, past, currently <15 g/day, currently ≥15 g/day	Question: ‘Do you have reduced hearing to such an extent that you feel it is difficult to follow a conversation between several people without using a hearing aid?’	Gender, age and smoking stratified by occupational noise exposure	7
Case-control study								
Jiao, 2017 [19]	China	Case 286/control 286	40.2	Both	Smoker, Non-smoker	Average hearing threshold ≥40 dB at 3, 4, 6 kHz	Gender, age, job category and time of exposure to noise	7
Jeffree, 2016 [28]	Malaysia	Case 49/control 98	41.3	Male	Smoking in pack-years: 0, 1–10, 11–20, >20	Average audibility threshold ≥25 dB at 0.5, 1, 2, 3 kHz	Daily noise dose, duration of services, HPD used frequency, perception about HPD	8
Cross-sectional study								
Wang,2017 [10]	China	11,196	67.1	Both	Smoking in pack-years: 0, 0–25, >25	Average audibility threshold ≥25 dB at 0.5, 1, 2, 4 kHz in both ears	Age, sex, race, shift work, occupational noise exposure, drinking status, hypertension, ototoxicity medicine, chronic diseases (diabetes mellitus, coronary heart disease, myocardial infarction and stroke)	10
Sari, 2017 [12]	Indonesia	122	18–40	Male	Smoker, Non-smoker	Average hearing threshold >25 dB at 0.5, 1, 2, 4 kHz	NA	6
Sriopas, 2017 [13]	Thailand	180	20–50	NA	Pack-years smoking: <10, ≥10	Average threshold >25 dB at 3, 4, 6, 8 kHz	Noise exposure level, employment duration, age, factory group, job position, and education level/noise exposure level, and education level	8
Win, 2015 [29]	Brunei	543	35.6	Both	Smoker, Non-smoker	Hearing loss of >25 dB at 4 kHz	NA	7
Sun, 2014 [45]	China	471	39.8	Male	Smoker, Non-smoker	Hearing any tone at 0.5, 1, 2 KHz >25 dB or average hearing threshold ≥40 dB at 3, 4, 6 kHz	Age, alcohol	9
Ferrite, 2013 [31]	Brazil	364	33.9	Female	Never smoked, Past smoker, Current smoker	Average threshold >25 dB at 0.5, 1, 2, 3, 4 kHz in the worse ear	Age, job type, solvent exposure and high blood pressure	10
Tao, 2013 [32]	China	517	37.9	Male	Smoker, Non-smoker	Hearing threshold >40 dB at 4 kHz in the worse ear	Age, CNE	8
Shen, 2013 [46]	China	495	40.6	Male	Smoker, Non-smoker	Average hearing threshold >40 dB at 3, 4, 6 kHz	Age, alcohol	8
Nasir, 2012 [33]	Malaysia	358	31.9	Both	Smoker, Non-smoker	Average hearing threshold ≥25 dB at 0.5, 1, 2, 3 kHz	Age, job type, Service duration, exposure duration, exposure to explosion, exposure to vibration	9
Shrestha, 2011 [7]	Nepal	110	29.8	Both	Smoker, Non-smoker	Average hearing loss >25 dB at 1, 2, 3 KHz	NA	6
Mohammadi, 2010 [34]	Iran	622	42.1	Male	Smoker, Non-smoker; Smoking in pack-years: 0, <20, ≥20	Average audibility threshold ≥25 dB at 0.5, 1, 2, 3 kHz	Age, duration of exposure	9
Attarchi, 2010 [35]	Iran	478	33.5	Male	Smoker, Non-smoker; Smoking in pack-years: 0, ≤8, >8	Hearing threshold differences ≥30 dB between 4 KHz and 1 KHz in both ears	Age, duration of exposure	8
Chang, 2009 [36]	China	75	42.4	Male	Smoker, Non-smoker	Average hearing loss >25 dB at 0.5, 1, and 2 kHz	Exposure status, age, tea or coffee, physical activity, BMI	8
Pouryaghoub, 2007 [37]	Iran	412	42.1	Male	Smoking in pack-years: 0, ≤10, >10	Hearing threshold >25 dB at 4 KHz in the better ear	Age, duration of exposure	8
Rachiotis, 2006 [38]	Greece	145	40.3	Both	Smoker, Non-smoker	Average threshold ≥25 dB at 4 KHz	Sex, age, occupational exposure to waste, duration of employment	8
Dement, 2005 [39]	USA	2469	56.6	Both	Smoker, Non-smoker	Index weighted average threshold >25 dB at 1, 2, 3, and 4 kHz	Age, race, and gender	9
Nomura, 2005 [41]	Japan	163	21–66	Male	Never smoked, Past smoker, Current smoker	Hearing loss >40 dB at 4 kHz	NA	7
Fortunato, 2004 [42]	Italy	94	43	Male	Smokers in cigarettes/day: ≤10, >10	Hearing any tone >25 dB	*PON2* (S/C) and *SOD2* IVS3-23 T/Gmand IVS3-60 T/G polymorphisms, age	8
Palmer, 2004 [20]	Britain	2232	16–64	Both	Never smoked, Past smoker, Current smoker	Question: ‘‘How well can you hear a person who is talking to you when he is sitting on your right [left] side in a quiet room?’’.	age, sex, and self report of frequent	8
Mizoue, 2003 [43]	Japan	1386	NA	Male	cigarettes/day: 0, 1–14, 15–24, ≥25	Hearing threshold >25 dB at 1 KHz and threshold > 40 dB at 4 KHz	Age	8
Barone, 1987 [44]	USA	1210	35.4	Male	Never smoked, Past smoker, Current smoker	Average hearing loss >25 dB at 1,2,3 KHz with a 5:1 weighting of the better to poorer ear	Age, years of present job	9

**Table 2:** Results of subgroup analysis between current smokers and NIHL risk.

Subgroup	Number of Studies	Pooled OR	95% CI	*P* Value for *Q* Test	*I^2^* (%)
Study design					
Cohort	4	1.19	1.10–1.28	0.504	0
Case-control	3	2.25	1.59–3.19	0.418	0
Cross-sectional	22	2.21	1.74–2.81	<0.001	89
Gender					
Both	13	1.50	1.28–1.76	<0.001	68
Male	14	3.05	1.90–4.89	<0.001	92
Female	1	1.52	1.03–2.27	-	-
Mean age					
<40	11	2.18	1.51–3.14	<0.001	86
≥40	16	2.03	1.59–2.61	<0.001	89
Race					
Mongoloid	16	1.88	1.50–2.36	<0.001	76
Caucasian	12	2.41	1.70–3.42	<0.001	93
others	1	1.52	1.03–2.27	-	-
Quality of studies					
High quality	24	2.14	1.73–2.64	<0.001	88
Moderate quality	5	1.91	1.05–3.45	<0.001	82
Number of adjusting variables					
0	4	1.58	0.86–2.90	<0.001	79
≥1	25	2.18	1.77–2.69	<0.001	88
Publication year					
<2010	12	2.27	1.53–3.34	<0.001	87
≥2010	17	1.90	1.53–2.36	<0.001	87

## Supplementary Materials

The following are available online at https://www.mdpi.com/1660-4601/17/4/1201/s1, Figure S1: Forest plot for the association between former smokers and NIHL risk, Figure S2: Sensitivity analysis of former smokers and NIHL, Figure S3: Funnel plot of current smokers and NIHL.
