# Supplementary material for: Association between Smoking and Noise-Induced Hearing Loss: A Meta-Analysis of Observational Studies
Source: Int J Environ Res Public Health. 2020 Feb 13;17(4):1201. doi: 10.3390/ijerph17041201 (PMC7068375; doi:10.3390/ijerph17041201)
Supplement: Supplementary file 1 [file ijerph-17-01201-s001.pdf]

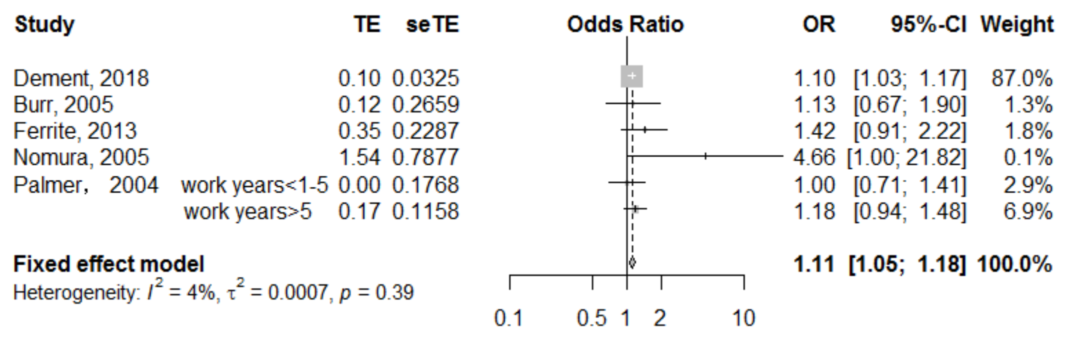

Figure S1. Forest plot for the association between former smokers and NIHL risk

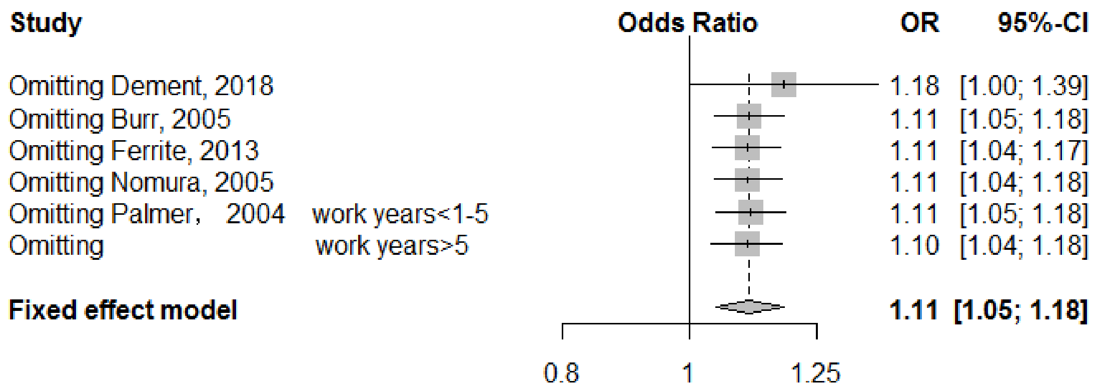

Figure S2. Sensitivity analysis of former smokers and NIHL

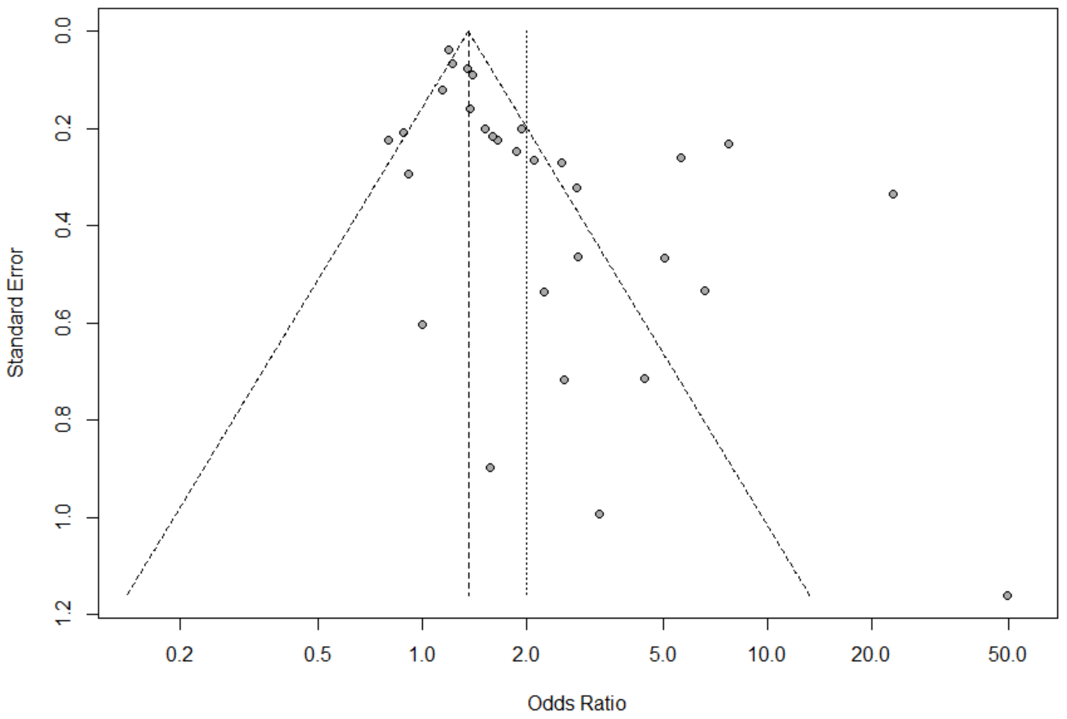

Figure S3. Funnel plot of current smokers and NIHL
